# Transcribed ultraconserved noncoding RNAs (T-UCR) are involved in Barrett's esophagus carcinogenesis

**DOI:** 10.18632/oncotarget.2249

**Published:** 2014-07-23

**Authors:** Matteo Fassan, Luigi Dall'Olmo, Marco Galasso, Chiara Braconi, Marco Pizzi, Stefano Realdon, Stefano Volinia, Nicola Valeri, Pierluigi Gasparini, Raffaele Baffa, Rhonda F. Souza, Caterina Vicentini, Edoardo D'Angelo, Jan Bornschein, Gerard J. Nuovo, Giovanni Zaninotto, Carlo M. Croce, Massimo Rugge

**Affiliations:** ^1^ Department of Medicine (DIMED), Surgical Pathology & Cytopathology Unit, University of Padua, Padua, Italy; ^2^ Department of Surgical Oncology and Gastroenterological Sciences (DiSCOG), University of Padua, Padua, Italy; ^3^ Comprehensive Cancer Center, Ohio State University, Columbus, OH; ^4^ Istituto Oncologico Veneto - IOV-IRCCS, Padua, Italy; ^5^ Department of Morphology and Embryology; University of Ferrara, Ferrara, Italy; ^6^ Institute of Cancer Research, London, UK; ^7^ Kimmel Cancer Center, Thomas Jefferson University, Philadelphia, PA; ^8^ Department of Medicine, University of Texas Southwestern Medical Center & VA North Texas Health Care System, Dallas, TX; ^9^ ARC-NET Research Centre, University of Verona, Verona, Italy; ^10^ Department of Gastroenterology, Hepatology and Infectious Diseases, Otto-von-Guericke-University of Magdeburg, Magdeburg, Germany; ^*^ Current address: Sanofi, Cambridge, MA, USA

**Keywords:** T-UCRs, Barrett's esophagus, Barrett's carcinogenesis, expression signature

## Abstract

Barrett's esophagus (BE) involves a metaplastic replacement of native esophageal squamous epithelium (Sq) by columnar-intestinalized mucosa, and it is the main risk factor for Barrett-related adenocarcinoma (BAc). Ultra-conserved regions (UCRs) are a class non-coding sequences that are conserved in humans, mice and rats. More than 90% of UCRs are transcribed (T-UCRs) in normal tissues, and are altered at transcriptional level in tumorigenesis. To identify the T-UCR profiles that are dysregulated in Barrett's mucosa transformation, microarray analysis was performed on a discovery set of 51 macro-dissected samples obtained from 14 long-segment BE patients. Results were validated in an independent series of esophageal biopsy/surgery specimens and in two murine models of Barrett's esophagus (i.e. esophagogastric-duodenal anastomosis). Progression from normal to BE to adenocarcinoma was each associated with specific and mutually exclusive T-UCR signatures that included up-regulation of uc.58-, uc.202-, uc.207-, and uc.223- and down-regulation of uc.214+. A 9 T-UCR signature characterized BE versus Sq (with the down-regulation of uc.161-, uc.165-, and uc.327-, and the up-regulation of uc.153-, uc.158-, uc.206-, uc.274-, uc.472-, and uc.473-). Analogous BE-specific T-UCR profiles were shared by human and murine lesions. This study is the first demonstration of a role for T-UCRs in the transformation of Barrett's mucosa.

## INTRODUCTION

Longstanding exposure of the esophageal mucosa to gastro-duodenal reflux is the primary risk factor for Barrett's esophagus (BE) [1-3]. Chronic inflammation of the esophageal mucosa results in its metaplastic replacement of the native squamous epithelia by columnar (intestinalized) cells. This cancer-prone epithelial population becomes the “cancerization field” in which intra-epithelial neoplasia (IEN, formerly called dysplasia) and Barrett-related esophageal adenocarcinoma (BAc) may develop [3-6].

The phenotypic changes from native to cancer mucosa result from a composite interaction of genetic dysregulations involving epigenetic silencing, transcription factors, signaling pathways and growth factors [7]. The biological machinery underlying the neoplastic transformation has yet to be fully elucidated, however.

In recent years, non-coding RNAs (ncRNAs) have generated a great deal of interest in cell transformation [7-9]. Among several families, ultraconserved regions (UCRs) were discovered in 2004 as a result of bio-informatic comparisons drawn between mouse, rat, and human genomes [10-13]. UCRs are composed of at least 481 genomic sequences more than 200 bp long (range 200-779 bp) that are absolutely conserved (100% identity with no insertions or deletions) among the three vertebrate species [10]. Single-nucleotide polymorphisms are under-represented in UCR genes, and these elements do not accumulate mutations in somatic cells under conditions of genomic instability [14-17]. A large fraction of UCRs are transcribed (T-UCRs) in normal human tissues, and their expression levels show both a ubiquitous and a tissue-specific pattern [14].

The function of T-UCRs is basically unknown, but their high trans-species conservation implies that they are fundamentally important to mammalian ontogenesis/phylogenesis. In their seminal work, Calin et al. [14] demonstrated that expression of T-UCRs are significantly altered at both DNA and RNA levels in adult chronic lymphocytic leukemias, as well as colorectal and hepatocellular carcinomas. They also found that tumor-associated T-UCRs are frequently located at fragile sites and cancer-associated genomic regions [14]. Recent genome-wide expression profiling studies have shown that T-UCRs exhibit distinct profiles in different human cancers, further supporting their role in human carcinogenesis [18-22].

This study is the first to demonstrate that a similar T-UCR expression profile in humans, rats and mice is associated with similar histological lesions involved in the morphogenesis of Barrett's adenocarcinoma. These results also support a pivotal involvement of specific T-UCRs in BE progression and recognize T-UCRs as a novel diagnostic/prognostic tool for the biological profiling of BE-associated lesions.

## RESULTS

### T-UCR expression is altered in Barrett's mucosa

**Figure 1:**
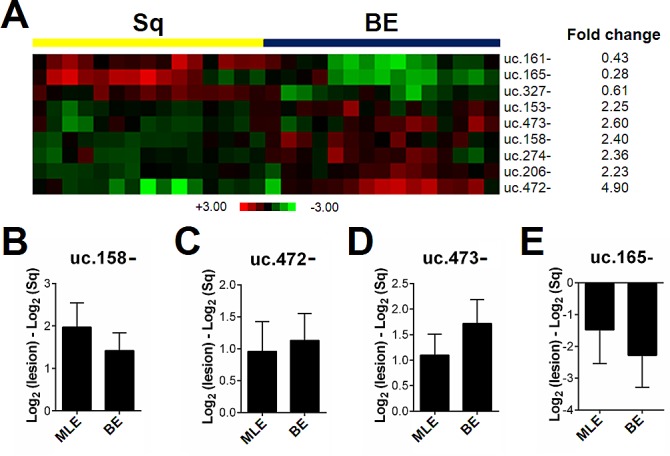
T-UCR expression is altered in esophageal metaplastic lesions (**A**) T-UCRs significantly dysregulated (p<0.001) in intestinal-type esophageal metaplasia (BE) by comparison with normal squamous esophageal epithelium (Sq). Rows represent individual T-UCRs; columns represent individual tissue samples. Pseudo-colors indicate transcript levels below, equating to, or above the mean (green, black, and red, respectively). The scale represents the intensity of gene expression (log2 scale ranges between -3 and +3). The up-regulation of uc.158- (**B**), uc.472- (**C**), and uc.473- (**D**), and the down-regulation of uc.165- (E) was confirmed in an independent series of biopsy samples for four of the nine dysregulated T-UCRs. The expression of the four T-UCRs was tested in multilayered epithelium (MLE), which is a pre-metaplastic phenotypic change, and showed a similar T-UCRs dysregulation to Barrett's mucosa (BE).

Epidemiological and clinico-pathological studies have consistently shown that BE is the initial event in a cascade of phenotypic changes that may lead to BAc. A 9 T-UCR signature (*p*<0.001) distinguished BE from squamous epithelium (Figure 1A). In particular, the BE signature disclosed three down-regulated (uc.161-, uc.165-, and uc.327-) and six up-regulated T-UCRs (uc.153-, uc.158-, uc.206-, uc.274-, uc.472-, uc.473-) (p<0.001). The up-regulation of uc.158- (Figure 1B), uc.472- (Figure 1C), and uc.473- (Figure 1D), and the down-regulation of uc.165- (Figure 1E) were confirmed by qRT-PCR analysis in an independent series of endoscopic biopsy samples obtained from 50 long-segment BE patients.

To further confirm the role of T-UCRs in the esophageal mucosa's acquisition of a metaplastic phenotype, 10 samples of MLE (recognized as an early-to-intermediate form of columnar metaplasia with both squamous and columnar features [23,24]) were included in the analysis. The BE and MLE samples generated similar results in the microarray data (Figure 1).

**Figure 2:**
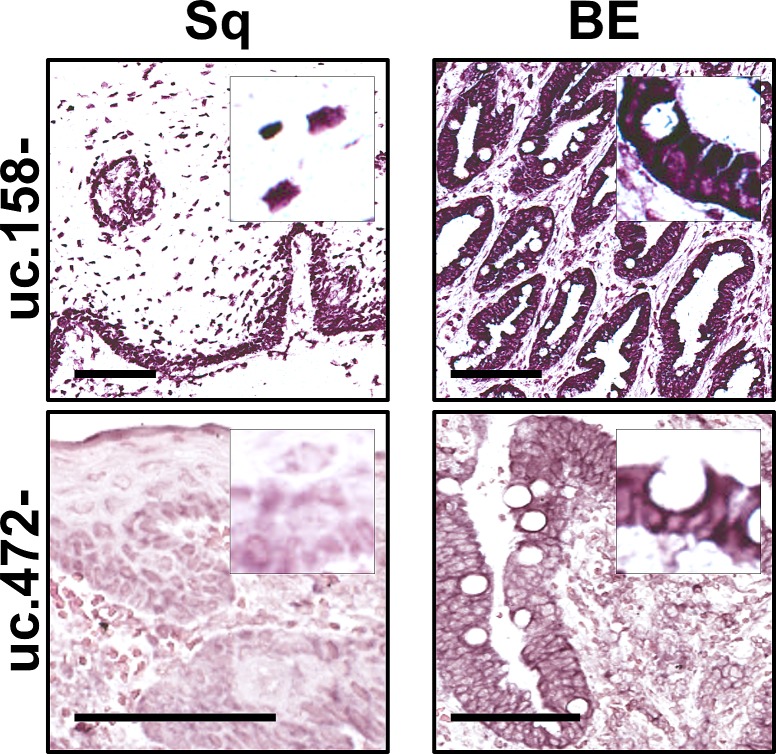
uc.158- and uc.472- are overexpressed in Barrett's mucosa Confirming microarray and qRT-PCR data, Barrett's mucosa consistently showed uc.158- and uc.472- overexpression by comparison with squamous esophageal epithelium on *in situ* hybridization analysis. Scale bars, 100 μm; original magnifications, 10x and 20x.

The up-regulation of uc.158- and uc.472- was confirmed by ISH in a series of 5 esophagectomy specimens (Figure 2). The two T-UCRs revealed both a nuclear and a cytoplasmic expression (with perinuclear reinforcement). Increased uc.158- expression was detected in 5 out of 5 BE specimens compared with matched Sq. As for uc.472-, this was moderately expressed in all 5 BE specimens, while Sq was weakly positive in the basal and suprabasal cell compartments.

### Barrett's mucosa T-UCR dysregulation is conserved across different species

**Figure 3:**
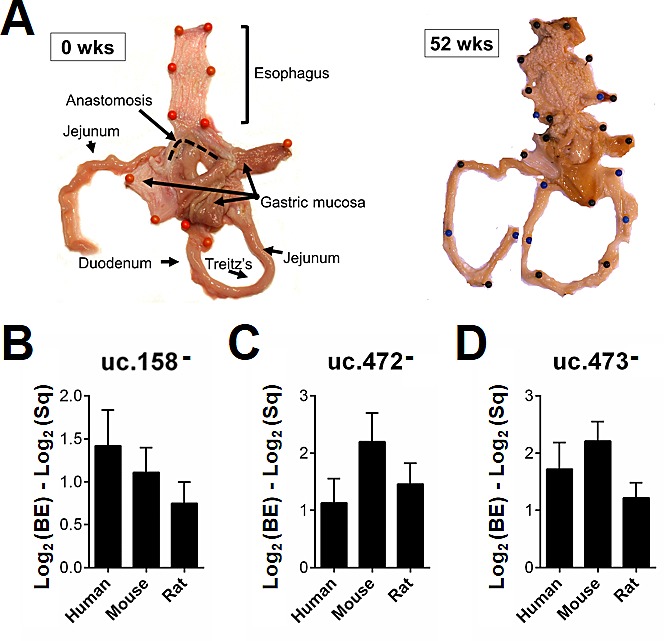
T-UCR dysregulation is comparable in human disease and murine models Five male Wistar Han rats and 5 male C57BL/6 mice underwent “Kumagai-Hattori” esophago-gastroduodenal anastomosis and were sacrificed 52 weeks after surgery. Their esophageal mucosa was macroscopically reddened with small protruding lesions (**A**, gross features representative of two surgically-treated rats). Areas of esophageal intestinal metaplasia (IM) were microdissected manually to assess T-UCR expression. An up-regulation of uc.158- (**B**), uc.472- (**C**), and uc.473- (**D**) was observed in the samples of IM from all three species considered.

Given that T-UCRs are conserved among humans, rats and mice [10-13], we developed two animal BE models based on the “Kumagai-Hattori” EGDA anastomosis [25] to investigate whether the T-UCR expression profiles in the animal models were akin to those seen in humans. Gross examination of 5 male Wistar Han rats and 5 male C57BL/6 mice sacrificed 52 weeks after surgery revealed a reddened esophageal mucosa with small protruding lesions in all cases (Figure 3A). All animals had reflux esophagitis proximal to the anastomosis. Mucosal ulcers were observed in the middle and lower thirds of the esophagus in 2/5 rats and 1/5 mice. Regenerative/hyperplastic changes were identified in 4/5 rats and 4/5 mice, and MLE and intestinal metaplasia (IM) in all cases. One rat had esophageal adenocarcinoma [25]. Areas of IM were manually microdissected to assess T-UCR expression. Three of the four T-UCRs tested (uc.158, uc.165, uc.472, and uc.473) showed a similar dysregulation in all three species considered (Figure 3B-D).

### T-UCR expression is altered in esophageal Barrett's adenocarcinoma

**Figure 4:**
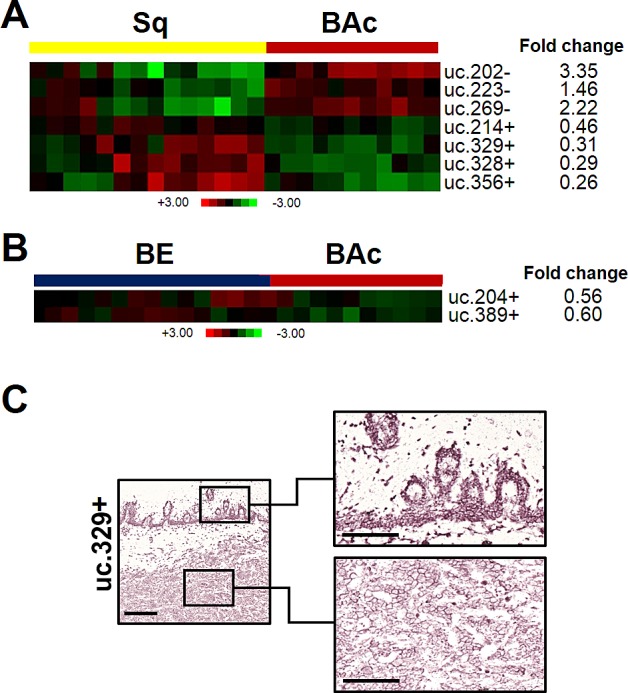
T-UCR expression is altered in Barrett's esophageal adenocarcinoma T-UCRs significantly dysregulated (p<0.001) in Barrett's adenocarcinoma (BAc) by comparison with normal squamous esophageal epithelium (Sq, **A**), or Barrett's mucosa (BE, **B**). Rows represent individual T-UCRs; columns represent individual tissue samples. Pseudo-colors indicate transcript levels below, equating to, or above the mean (green, black, and red, respectively). The scale represents the intensity of gene expression (log2 scale ranges between -3 and 3). (**C**) ISH analysis confirmed that uc.329+ was significantly down-regulated in BAc. Sq showed moderate uc.329+ expression in basal and suprabasal cell compartments (right, upper panel); which was much decreased in the BAc. Scale bars, 200 and 100 μm; original magnifications, 5x and 20x.

Different T-UCR expression profiles were identified when Sq and BE were compared with BAc (Figure 4A-C). In particular, seven T-UCRs were found dysregulated in BAc by comparison with Sq (uc.202-, uc.223-, and uc.269- were up-regulated, and uc.214+, uc.328+, uc.329+, and uc.356+ were down-regulated) and two were dysregulated in BAc by comparison with BE (uc.204+ and uc.389+ were down-regulated). ISH confirmed the down-regulation of uc.329+ in BAc in 4/5 cases (Figure 4B). The uc.329+ expression was mainly cytoplasmic. A moderate expression was observed in the basal and suprabasal cell compartments of all five Sq specimens.

### T-UCR expression is altered in the carcinogenic progression from squamous epithelium to Barrett's adenocarcinoma

To identify the T-UCR profiles that are dysregulated in Barrett's mucosa, T-UCR microarray analysis was performed on a discovery set of 51 macro-dissected samples obtained from 14 patients with long-segment BE who had undergone esophagectomy. The lesions considered were representative of the whole phenotypic spectrum of lesions observed in Barrett's carcinogenesis, with 14 samples of Sq, 14 of BE, 7 of LG, 5 of HG, and 11 of BAc. T-UCR microarray analysis was performed using a validated custom microarray platform [14].

**Figure 5:**
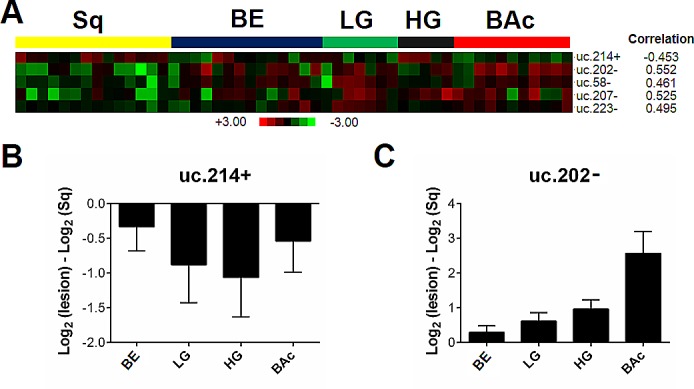
T-UCR expression is altered in the carcinogenic progression from squamous epithelium to Barrett's adenocarcinoma (**A**) T-UCRs significantly dysregulated in the Barrett's carcinogenic cascade (normal squamous epithelium [Sq] - Barrett's mucosa [BE] - low-grade intra-epithelial neoplasia [LG] - high-grade intra-epithelial neoplasia [HG] - Barrett's adenocarcinoma [BAc]) as assessed on microarray data. Rows represent individual genes; columns represent individual tissue samples. Pseudo-colors indicate transcript levels below, equating to, or above the mean (green, black, and red, respectively). The scale represents the intensity of gene expression (log2 scale ranges between -3 and 3). The down-regulation of uc.214+ (**B**) and the up-regulation of uc.202- (**C**) were confirmed by qRT-PCR in a separate set of biopsy samples.

The analysis identified five T-UCRs that were differently expressed (*p*<0.001) in cases undergoing Barrett's carcinogenesis (Figure 5A). In particular, the signature included four T-UCRs (uc.58-, uc.202-, uc.207-, and uc.223-) with a higher expression and one (uc.214+) with a lower expression. Three of the Sq-BAc dysregulated T-UCRs (i.e. uc.202-, uc.214+ and uc.223) were shared with the progression signature from Sq to BAc (Figure 5A). QRT-PCR analyses for uc.214+ (Figure 5B) and uc.202- (Figure 5C) confirmed the microarray data.

The chromosomal distribution of the T-UCRs dysregulated during Barrett's carcinogenesis indicated that chromosomes 5, 7, 9, 11 and X were the most involved in this process. In particular, there were three down-regulated T-UCRs (uc.327-, uc.328+, and uc.329+) located at 11p13, two up-regulated T-UCRs (uc.206-, uc.207-) at 7p15.3, and another two up-regulated T-UCRs (uc.269-, uc.274-) at 9q33.3.

## DISCUSSION

Only fragmentary information is available on the molecular changes driving the phenotypic shift from native squamous esophageal epithelium to metaplastic Barrett's mucosa, and to the latter's neoplastic transformation. The fact that no well-established biomarkers of disease progression are available currently prevents any molecular monitoring of Barrett's disease or molecular-based strategies for the secondary prevention of BAc [7,8].

It has recently been established non-coding RNAs as key-role players molecules in human carcinogenesis and several different new classes of non-coding RNAs (e.g. miRNAs, T-UCRs, lincRNAs, and snoRNAs) have been identified [26,27]. Previous findings identified a pivotal function of miRNA dysregulation in generating the Barrett's phenotype and driving its neoplastic transformation [8,9,24,28].

Ultraconserved regions (UCRs) form a subset of conserved sequences located in both intra- and inter-genic regions [22]. They are absolutely conserved (100%) between orthologous regions of the human, rat and mouse genomes, and they exhibit almost no natural variation in the human population [14]. The majority (93%) of UCRs are transcribed (T-UCRs) in normal human tissues, both ubiquitously and in a tissue-specific manner [14]. Most importantly, recent data suggest that T-UCRs are altered at transcriptional level in human tumorigenesis and aberrant T-UCR expression profiles may discriminate between different human cancers. Like known cancer-related genes, some T-UCRs have been found to undergo CpG island hypermethylation-associated silencing [10]. Only very few T-UCRs have been described functionally *in vitro*, however: uc.73, for example, was found to influence apoptosis in colon cancer cells, and uc.338 to inhibit the growth of hepatocellular carcinoma cells [19,20].

Different putative functions have been hypothesized for T-UCRs, including an antisense inhibitory role in protein coding genes or other non-coding RNAs [14]. Although UCRs are significantly depleted among segmental duplications and copy-number variants [29], the deletion of some of these regions in knock-out mice was not associated to any notable phenotypic abnormality [30]. Most recently, T-UCRs have been implicated: (i) in long-range enhancer-like activities; (ii) in the homeostatic maintenance of splicing factor expression levels; and (iii) in regulating transcription, both as epigenetic modification marks, and as transcriptional co-activators [31-38].

Here, we examined the expression of transcripts encoded by the 481 UCR genes in Barrett's carcinogenesis. The present findings demonstrate that: (i) T-UCR dysregulation occurs early in BE morphogenesis; (ii) similar T-UCR dysregulations are found in both human and murine intestinalized columnar metaplasia, and this supports a “basic” biological consistency between T-UCR-related oncogenic pathways; (iii) T-UCR signatures show a “progressive” dysregulation along the path from squamous epithelia to adenocarcinoma, supporting their oncogenic role in Barrett's carcinogenesis.

The T-UCR profiles that we obtained are unlike those previously reported in other types of human cancer (e.g. leukemia, colon cancer, liver cancer, prostate cancer, and neuroblastoma) [18-22,39,40] and this underscores the fact that T-UCR expression is specific to a given tissue and cancer histotype, reminiscent of the miRNA expression patterns described in human solid tumors [41].

The clustering of dysregulated T-UCRs in specific chromosome loci (7p15.3, 9q33.3, and 11p13) previously described as being deleted and/or amplified in Barrett-related lesions [42-45] points to genome instability having a significant influence on T-UCR expression profiles. Comparative genomic hybridization analysis was not feasible in the present study due to the limited size of the pre-neoplastic lesions within BE mucosa considered, and most of the material available was needed for T-UCR profiling.

Having identified similar T-UCR profiles in different species points to T-UCRs having a core role in the acquisition of the pre-neoplastic and neoplastic phenotypes involved in the cascade of Barrett's carcinogenesis. This study was the first to investigate the T-UCR profiles associated with the natural history of a given disease in three different mammal species, and the results obtained support the trans-species involvement of T-UCRs as molecular drivers of cell homeostasis in different vertebrates.

Further (microarray and *in vitro)* studies should investigate the influence of chromosomal instability on T-UCR expression and the functional role of T-UCRs in oncogenesis. Our results can also serve as a starting point for further studies on this still little-explored non-coding RNA family as biomarkers of BE-associated cancer risk.

## MATERIAL AND METHODS

### Patients

A first discovery set used in the T-UCR microarray study concerned 14 BE patients who had undergone esophagectomy for high-grade intra-epithelial neoplasia (HG) and BAc (mean age 63.6±7.9 years, range 52-81; 12 males, 2 females; all Caucasian). Two 2 mm cores were obtained from the paraffin blocks from: (a) the proximal native squamous esophageal mucosa (Sq=14 cases; ≥ 3 cm far from any BE); (b) IM-positive Barrett's mucosa (BE= 14 cases); (c) low-grade intra-epithelial neoplasia (LG= 7 cases); (d) high-grade intra-epithelial neoplasia (HG= 5 cases); and (e) BAc (11 cases).

A validation set used in the qRT-PCR study consisted of 60 biopsy samples obtained from 50 patients with histologically-proven long-segment BE (mean age 64.4±8.7, range 54–76; 40 males, 10 females; all Caucasian). Cases were retrospectively collected from the files of the Veneto Region's multicenter Barrett's Esophagus Registry (EBRA [www.esofagodibarrett.it]; Padua Unit) [46]. All patients had endoscopically-confirmed long-segment BE (≥ 3 cm) and were biopsied according to the Seattle protocol (i.e. four-quadrant biopsies were obtained from every 2 cm of metaplastic mucosa). Biopsy samples were collected from paraffin blocks and included: Sq= 10 cases, BE= 10 cases, LG= 10 cases, HG= 10 cases, BAc= 10 cases. Ten further samples of multilayered epithelium (MLE) were also considered. MLE is defined as multilayered, flattened squamoid epithelium overlaid by columnar mucus-producing, non-intestinalized cells, and has been proposed as an early precursor of Barrett's metaplastic transformation [23,24]. Five BAc esophagectomy specimens were used in the *in situ* hybridization study.

Original slides or serial sections (4-6 microns thick) obtained from archival paraffin-embedded tissue samples (hematoxylin & eosin [H&E], Alcian-PAS) were jointly re-assessed by two GI pathologists (MF and MP). If their opinions differed, a third expert GI pathologist (MR) was consulted. The institute's ethical regulations concerning research conducted on human tissues were followed, and all patients considered in this study gave their written informed consent.

### T-UCR microarray

Formalin-fixed, paraffin-embedded samples underwent total RNA extraction using the RecoverAll kit according to the manufacturer's instructions (Ambion Inc., Austin, TX). RNA labeling and hybridization on microarray chips were done as described in detail elsewhere [14]. Briefly, 5 μg of total RNA from each sample were reverse-transcribed using biotin end-labeled random-octamer oligonucleotide primer. Biotin-labeled complementary DNA was hybridized on an Ohio State University custom miRNA microarray chip (OSU_CCC version 4.0) developed with a total of 481 human UCR sequences [14]. For each UCR, two 40-mer probes were designed, one corresponding to the sense genomic sequence (named ‘+’) and the other to the complementary sequence (named ‘-’). Each oligo was printed in duplicate at two different slide locations, so quadruplicate numerical values were available for analysis. The hybridized chips were washed and processed for biotin-containing transcript detection with streptavidin-Alexa 647 conjugate and scanned on an Axon 4000B microarray scanner (Axon Instruments, Sunnyvale, CA). Microarray images were analyzed using GENEPIX PRO 6.0 (Axon Instruments). Average values of the replicate spots of each T-UCR were background subtracted, normalized using quantiles to enable a comparison between chips, and further analyzed. The microarray data are deposited in the Gene Expression Omnibus at the National Center for Biotechnology Information [GEO: GSE20099].

### Murine models of BE

This study was approved by the Institutional Animal Care Committee of the University of Padua. All procedures were performed in accordance with Italian law on the use of experimental animals (DL n. 116/92 art. 5) and according to the “Guidelines on the Care and Use of Laboratory Animals” (NIH publication 85-93, revised in 1985).

A “Kumagai-Hattori” esophagogastric-duodenal anastomosis (EGDA) was performed on 6 eight-week-old male Wistar Han rats and 6 eight-week-old male C57BL/6 mice (Charles River, Lecco, Italy), as described elsewhere [25]. Briefly, a side-to-side surgical EGDA was created between the first duodenal loop and the gastro-esophageal junction, with accurate mucosa-to-mucosa opposition, so that duodenal and gastric contents flowed back into the esophagus. After 30 weeks from surgery, animals usually present numerous patchy MLE-like and BE-like lesions across the entire esophageal and upper gastric mucosa.

Postoperatively, the animals had free access to water and food. No treatments with any known carcinogenic potential were applied. One rat and one mouse died within 7 days after surgery and were not considered. The animals were sacrificed 52 weeks after surgery and, immediately afterwards, the esophagus was opened longitudinally through the dorsal wall. With the mucosal surface uppermost, the margins of the specimen were attached to a cork plate with pins. Gross specimens were fixed in 10% neutral-buffered formalin for 24 hours. All specimens were examined grossly and cut serially (2–3 mm thick coronal sections). Lung, liver, kidney and spleen tissues were also collected for histology.

### Quantitative real-time polymerase chain reaction

Formalin-fixed, paraffin-embedded samples were manually micro-dissected to obtain 70% of pre-neoplastic or neoplastic cells, then deparaffinized before undergoing total RNA extraction using the RecoverAll kit according to the manufacturer's instructions (Ambion Inc., Austin, TX). T-UCRs were quantified by means of pre-optimized T-UCR real-time PCR assays (PrimerDesign Ltd, Hants, UK) using SYBR® green chemistry. Each assay was validated individually by PrimerDesign Ltd and found 100% specific and nearly 100% efficient. Normalization was done with the small nuclear U6 RNA (Exiqon). Comparative real-time PCR was run in triplicate, including no-template controls. The fold difference for each sample was obtained using the ΔΔCT method.

### *In situ* RNA hybridization (ISH)

Locked nucleic acid (LNA) probes complementary to the 20-22-bp sections of uc.158, uc.329, and uc.472 were labeled with 5′-digoxigenin and synthesized by Exiqon (Denmark). Tissue sections were digested with ISH protease 1 (Ventana Medical Systems, Milan, Italy) and ISH was performed as described [20]. Negative controls included omission of the probe and the use of a scrambled LNA probe.

### Statistical analysis

For microarray studies T-UCRs that were differently expressed in different esophageal lesions were identified using a random-variance *t*-test. Genes were considered statistically significant if their *p* value was less than 0.001 (such a stringent significance threshold was used to limit the number of false positive findings). A linear regression model (using normalized log2-transformed expression values) was applied to test significant dysregulated T-UCRs in the different lesions, and *p*-values were adjusted for multiple testing using false discovery rate (FDR) correction (only FDRs with a *p*<0.0001 were considered). Differences between groups in qRT-PCR analysis were tested by applying the *t-*test or the Pearson's correlation test. *P* values less than 0.05 were considered significant. The statistical analysis was performed using STATA 8.0 software (Stata Corporation, College Station, TX).

## List of abbreviations

T-UCR: transcribed ultraconserved regions
miRNA: microRNA
BE: Barrett's esophagus
Sq: native stratified squamous epithelium
LG: low-grade intra-epithelial neoplasia
HG: high-grade intra-epithelial neoplasia
BAc: Barrett-related esophageal adenocarcinoma
IM: intestinal metaplasia
